# A simple method for discrimination of carcinomatous meningitis using CEA, total protein, and total cell count in the cerebrospinal fluid of primary lung cancer patients

**DOI:** 10.1097/MD.0000000000025367

**Published:** 2021-04-09

**Authors:** Yukari Ogawa, Takeshi Saraya, Akinari Noda, Nozomi Kurokawa, Sho Sakuma, Kaori Aso, Sunao Mikura, Miku Oda, Manabu Ishida, Kojiro Honda, Keitaro Nakamoto, Masaki Tamura, Saori Takata, Haruyuki Ishii, Hajime Takizawa

**Affiliations:** Department of Respiratory Medicine, Kyorin University School of Medicine, 6-20-2 Shinkawa, Mitaka City 181-8611, Tokyo, Japan.

**Keywords:** carcinoembryonic antigen, cerebrospinal fluid, prognosis, total protein

## Abstract

Carcinomatous meningitis (CM) is a critical issue for physicians. However, no study has reported a simple and useful diagnostic or predictive marker for CM.

This study aimed to elucidate the potential markers for diagnosing CM derived from cerebrospinal fluid (CSF).

We retrospectively enrolled 78 lung cancer patients with suspected CM during the clinical course, including 42 CM and 36 non-CM patients. We compared the clinical and CSF findings, including carcinoembryonic antigen (CEA), between CM and non-CM patients, and explored the diagnostic markers for early identification of CM as well as the contributing factors for mortality.

On CSF analysis, with cutoff values of CEA ≥5 ng/ml, total protein (TP) in CSF ≥45 g/dl, and total cell count (TCC) ≥7 cells/μL, the sensitivity, specificity, and area under the curve (AUC) for CM were 85.7%, 84.6%, and 0.887 (95% CI: 0.758–1.0, *P* < .001); 80.5%, 69.4%, and 0.755 (95% CI: 0.646–0.865, *P* < .001); and 56.1%, 100%, and 0.817 (95% CI: 0.722–0.912, *P* < .001), respectively. TP levels in CSF ≥the patients’ age had a sensitivity, specificity, and an AUC of 48.8%, 77.8%, and 0.633 (95% CI: 0.722–0.912, *P* = .045) for CM, respectively. Among CM patients, patients with ‘TP in CSF (>patients’ age)” (n = 19, *P* = .008) showed significantly shorter 90-day survival probability than the residual patients (n = 20). None of the CSF parameters could predict the risk of mortality on Cox regression analysis.

The cutoff value of CEA ≥5 ng/ml in CSF is a simple and useful method with a high diagnostic value for CM diagnosis, but not a suitable predicting factor for mortality. ‘TP in CSF >patients’ age” might be a novel factor for assessing short-term mortality.

## 1 Introduction

The incidence of brain metastasis in patients with primary lung cancer accounts for up to 20.6% of all metastases in their clinical course.^[1]^ However, the frequency of carcinomatous meningitis (CM) in lung cancer is considered to be approximately 1.4%,^[2]^ and the consensus for CM diagnosis specifically focusing on the cerebrospinal fluid (CSF) findings has been scarcely reported. In this modern era, evolutionarily developed treatments have been attributed to prolonged survival, even in the setting of CM. Therefore, a simple and useful diagnostic tool for CM is urgently required.

## 2 Material and methods

### 2.1 Study design

We retrospectively enrolled all patients with lung cancer who were suspected of having CM manifesting symptoms (headache, altered consciousness, unstable gait, nausea, vomiting, incontinence, weakness) or signs (cranial nerve involvement, mental changes, cerebellar signs, and lower motor neuron deficits). They had benefited from a lumbar puncture at the Kyorin University Hospital, a 1100-bed capacity tertiary center located in the west of Tokyo from January 2012 to December 2019.

### 2.2 Definition of CM

The definition of CM is as follows:

1. Results of CSF cytology were class IV or V.
2. Characteristic radiological features on enhanced magnetic resonance imaging revealed diffuse enhancement in the cerebral sulcus/cistern or surface of the cerebellum, enhanced nodule in the subarachnoid space or brain ventricle, abnormal enhancement of the ventricular wall, expansion of the ventricular space without tumor occlusion, and enhancement of the meninges.
3. Abnormal CSF findings included an elevation of total protein (TP) ≥45 g/dl or total cell count (TCC) ≥5 cells/μL, and opening pressure at the lumbar spine of 18 cm H_2_0.

**Figure 1:**
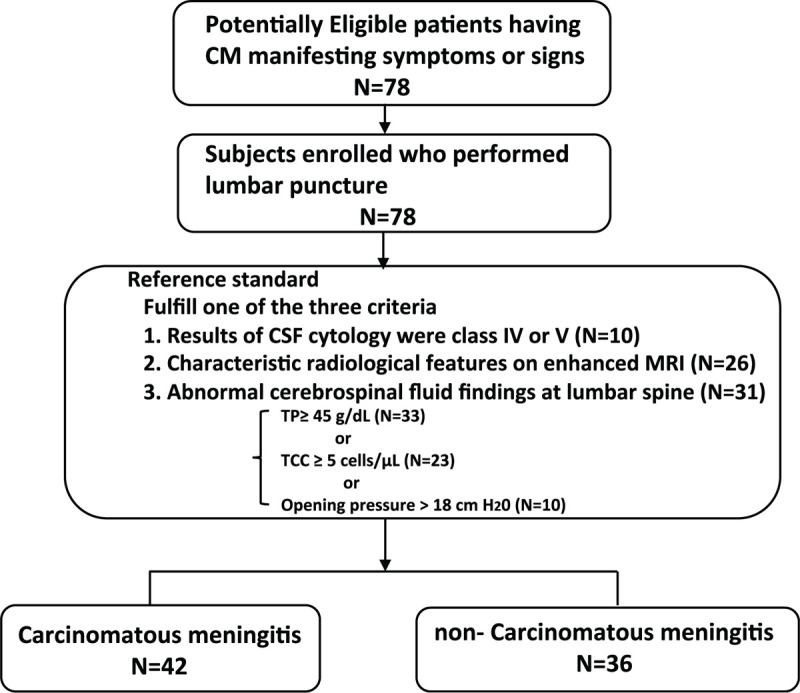
Study flowchart. CSF = cerebrospinal fluid, MRI = magnetic resonance imaging, TCC = total cell count, TP = total protein.

Patients who fulfilled one of the above-mentioned 3 criteria were considered as having CM, while the other residual patients were defined as non-CM patients. A total of 78 patients with suspected CM who underwent lumbar puncture were finally enrolled, comprise of CM (n = 42) and non-CM patients (n = 36) (Fig. 1)

### 2.3 Discrimination of CM and non-CM patients

We compared the characteristics of CM and non-CM patients such as age, sex, previous treatment regimens, type of lung cancer, and CSF findings including carcinoembryonic antigen (CEA). We examined the data to explore the appropriate threshold for CEA, TP, and TCC in CSF.

### 2.4 Predicting factors for survival probabilities in CM patients

We examined the factors affecting the survival probability using various CSF parameters and other clinical findings.

### 2.5 Statistical analysis

Numerical data were evaluated for normal distribution and equal variance using the Kolmogorov–Smirnov test and Levene median test, respectively. Categorical data were presented as percentages of the total or numerically, as appropriate. Statistical comparisons of non-parametric data were performed using the Mann–Whitney test. Categorical data were compared using Pearson Chi-Squared test. Logistic regression modeling was used for univariate and multivariate analyses to identify risk factors for CM. Receiver-operator characteristic (ROC) curves defining the sensitivity and specificity for diagnosing CM were constructed for the CSF parameters (CEA, TP, and TCC). All tests were two-sided, and significance was indicated by values of *P* < .05. Data were analyzed using SPSS version 25.0 software for Windows. This study was approved by the ethical committee of Kyorin University Hospital (H30-189).

## 3 Results

### 3.1 Discrimination of CM and non-CM patients

#### 3.1.1 Clinical characteristics between patients with or without CM

We enrolled a total of 78 patients with primary lung cancer, comprising 42 CM patients and 36 non-CM patients in the course of the disease (Fig. 1). Patient characteristics such as age, sex, previous treatment regimens, and type of lung cancer were comparable between the CM and non-CM groups (Table 1). The CSF analysis demonstrated that the values (median; min-max) of TCC, TP, and CEA were significantly higher in the CM group (8.0, 1.0–105 cells/μL, *P* < .001) (68.7, 29.3–2461 g/dl, *P* < .001) (37.6, 0.5–2290 ng/ml, *P* < .001) than in the non-CM group (1.0, 0–6.0) (39.6, 21.8–1637 g/dl) (0.5, 0.5–48.7 ng/ml), respectively. Interestingly, the proportion of TP in CSF (>patients’ age) was significantly greater in the CM group (48.8%, n = 20, *P* = .014) than in the non-CM group (22.2%, n = 8).

**Table 1:** Clinical characteristics between patients with or without carcinomatous meningitis.

Carcinomatous meningitis	YES (N = 42)	NO (N = 36)	*P* value
Age	67 (50–85)	70 (41–84)	.215
Sex (Male/Female)	27/15	24/12	1.0
Previous regimens of treatments	2.0 (0–7.0)	2.0 (0–6.0)	.078
Type of lung cancer			
adeno	31	24	.619
small	9	7	1.0
other	2	5	.239
CSF findings			
Opening pressure (cmH2O)	14.0 (4.0–40.0)	12.0 (4.5–23.5)	.054
Total cell counts (cells/μL)	8.0 (1.0–105)	1.0 (0–6.0)	<.001
Total protein (g/dl)	68.7 (29.3–2461)	39.6 (21.8–1637)	<.001
Total protein (>patients’ age)	48.8% (n = 20)	22.2% (n = 8)	.014
Glucose (mg/dl)	53.5 (14.0–154)	61.5 (7.0–134)	.067
CEA (ng/ml)	37.6 (0.5–2290)	0.5 (0.5–48.7)	<.001
Serum CEA (ng/ml)	95.9 (2.0–7303)	20.6 (1.1–2581)	.138
CNS (+/−)	18/24	16/19	.822
EGFR mutation (+)/number of exam	15/30	10/26	.430

All data are expressed as median (min - max), CEA = Carcinoembryonic antigen, CNS = central nervous system, CSF = cerebrospinal fluid, EGFR = epidermal growth factor receptor.

Serum CEA levels, the proportion of CNS metastasis, and the positive ratio of epidermal growth factor receptor (EGFR) mutation was comparable in both groups.

#### 3.1.2 Discrimination between the patients with or without CM using CEA, TP, and TCC in CSF analysis

**Figure 2:**
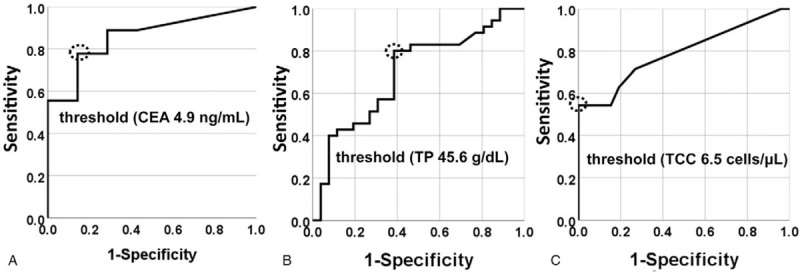
ROC curve for discrimination of CM showed that the preferable thresholds of CEA, TP, and TCC in CSF were 4.9 ng/ml (Fig. 2A 2A45.6 g/dl (Fig. 2B), and 6.5 cells/μL (Fig. 2C), respectively.

Based on the data of patients’ backgrounds, we compared the CEA concentrations, TP, and TCC levels in CSF between CM and non-CM patients. The thresholds of CEA, TP, and TCC in CSF were 4.9 ng/ml (Fig. 2A), 45.6 g/dl (Fig. 2B), and 6.5 cells/μL (Fig. 2C). If the cutoff value for CEA was set at ≥5 ng/ml, the sensitivity and specificity were 85.7% and 84.6%, respectively, with an area under the curve (AUC) of 0.887 (95% CI: 0.758–1.0, *P* < .001). In addition, TP in CSF ≥ 45 g/dl showed a sensitivity of 80.5% and specificity of 69.4% with an AUC of 0.755 (95% CI: 0.646–0.865, *P* < .001), and TCC ≥7 cells/μL showed a sensitivity of 56.1% and specificity of 100% with an AUC of 0.817 (95% CI: 0.722–0.912, *P* < .001) (Table 2). Furthermore, “TP in CSF (> patients’ age)” had a sensitivity of 48.8% and specificity of 77.8% with an AUC of 0.633 (95% CI: 0.722–0.912, *P* = .045). The combinations of parameters such as “CEA≧5.0 (ng/ml) and TP≧45 (g/dl),” “CEA≧5.0 (ng/ml) and TCC ≧7 (cells/μL),” and “CEA≧5.0 (ng/ml) and TP in CSF (>patients’ age)” were reliable diagnostic factors, with their sensitivity, specificity, AUC, 95%CI, and significance being 78.6%, 100%, 0.893, 0.758 to 1.0 (*P* = .001); 64.3%, 100%, 0.821, 0.654 to 0.989 (*P* = .005); and 64.3%, 92.3%, 0.783, 0.602–0.964 (*P* = .012), respectively (Table 2).

**Table 2:** Proposal cutoff values in CSF for discrimination of carcinomatous meningitis.

	Sensitivity	Specificity	PPV	NPV	PLR	NLR	AUC	95%CI	*P* value
CEA≧5.0 (ng/ml)	85.7	84.6	85.7	84.6	5.56	0.17	0.887	0.758–1.0	<.001
TP ≧45 (g/dl)	80.5	69.4	75	75.8	2.63	0.28	0.755	0.646–0.865	<.001
TP in CSF (>patients’ age)	48.8	77.8	71.4	57.1	2.19	0.65	0.633	0.508–0.758	.045
TCC ≧7 (cells/μL)	56.1	100	95.8	66.0	0	0.44	0.817	0.722–0.912	<.001
CEA≧5.0 (ng/ml)and TP≧45 (g/dl)	78.6	100	78.6	100	0	0.21	0.893	0.758–1.0	.001
CEA≧5.0 (ng/ml) and TP in CSF (>patients’ age)	64.3	92.3	90.0	70.6	8.35	0.39	0.783	0.602–0.964	.012
CEA≧5.0 (ng/ml) and TCC ≧7 (cells/μL)	64.3	100	100	72.2	0	0.36	0.821	0.654–0.989	.005
TP ≧45 (g/dl) and TCC ≧7 (cells/μL)	48.8	100	100	63.6	0	0.51	0.744	0.633–0.855	<.001
TP in CSF (>patients’ age) and TCC ≧7 (cells/μL)	36.6	100	100	58.1	0	0.63	0.683	0.564–0.802	.006

AUC = area under the curve, CEA = carcinoembryonic antigen, CSF = cerebrospinal fluid, NLR = negative likelihood ratio, NPV = negative predictive value, PLR = positive likelihood ratio, PPV = positive predictive value, TCC = total cell count, TP = total protein.

### 3.2 Difference of survival probabilities in CM patients

#### 3.2.1 The 90-day survival probabilities on Kaplan–Meier plot according to the factors of TP levels in CSF (>patients’ age) and EGFR mutation

**Figure 3:**
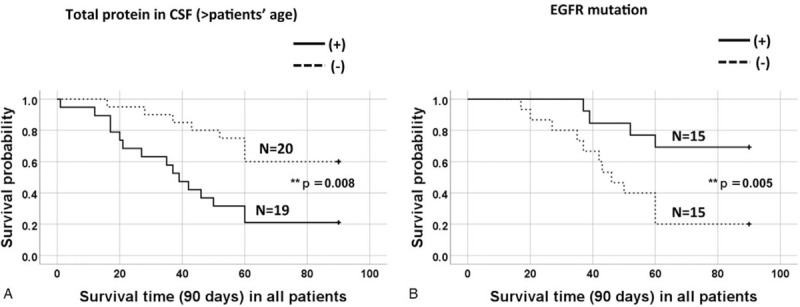
On the Kaplan–Meier plot, if the TP in CSF is greater than patient's age, the 90-day survival probability was significantly lower (n = 19, *P* = .008) than that of the residual patients (n = 20) (Fig. 3A 3A). If the patients have EGFR mutation, the 90-day survival probability was significantly higher in the EGFR mutation-positive patients (n = 15, *P* = .005) than in the negative ones (n = 15) (Fig. 3B).

Among the CM patients (n = 42), we successfully retrieved the CSF data of only 39 patients. If the TP levels in the CSF were equal to or higher than that of the patients’ age, the 90-day survival probability was significantly lower (n = 19, *P* = .008) than that of the residual patients (n = 20) (Fig. 3A). If the patients had EGFR mutations, the 90-day survival probability was significantly higher in EGFR mutation-positive patients (n = 15, *P* = .005) than in the negative patients (n = 15) (Fig. 3B). Among these 2 groups, the ratio of further treatment after the diagnosis of CM was significantly higher in the former group (n = 11, *P* = .003) than in the latter group (n = 2). The treatment regimens in the EGFR mutation-positive group were tyrosine kinase inhibitors (TKIs) (erlotinib, n = 6; gefitinib, n = 1; afatinib, n = 1), followed by other intravenous chemotherapies (n = 3), and only 2 patients with EGFR mutation-negative were treated with docetaxel.

Among the patients who did not undergo EGFR mutation testing (n = 12), only 2 patients were treated with intravenous chemotherapies such as carboplatin plus paclitaxel or amrubicin hydrochloride.

### 3.3 Predictive factors for CM

#### 3.3.1 Univariate Cox regression analysis associated with the risk of mortality in CM

Based on the univariate Cox regression analysis, predictive factors for mortality among CM patients were as follows: TP levels in the CSF were higher than the patients’ age (HR 2.47, 95% CI, 1.26.4.82, *P* = .008), receiving the best supportive care after the diagnosis of CM (HR 6.12, 95% CI: 2.61–14.3, *P* < .001), and being EGFR mutation-negative (HR 3.63, 95% CI: 1.49–8.89, *P* = .005) (Table 3). However, none of these factors, TP ≥45 g/dl or TCC ≥7 cells/μL, and CEA ≥5 ng/ml in CSF, can be predictive factors for mortality (Table 3).

**Table 3:** Univariate cox regression analysis associated with the risk of mortality in carcinomatous meningitis.

CSF	HR	95% CI	*P* value
TP (>patients’ age)	2.47	1.26–4.82	.008
CEA ≧5 (ng/ml)	2.96	0.36–24.0	.310
TCC ≧7 (cells)	1.59	0.81–3.10	.171
TP ≧45 (g/dl)	2.36	0.97–5.74	.059
No treatment after diagnosis of CM	6.12	2.61–14.3	<.001
CNS metastasis at the diagnosis of lung cancer	1.17	0.62–2.20	.629
EGFR mutation negative	3.63	1.49–8.89	.005

CEA = carcinoembryonic antigen, CM = carcinomatous meningitis, CNS; central nervous system, EGFR = epidermal growth factor receptor, HR = hazard ratio, TCC = total cell count, TP = total protein.

#### 3.3.2 Multivariate Cox regression analysis associated with the risk of mortality in CM

On multivariate Cox regression analysis, a negative EGFR mutation was the sole predictive factor for mortality (HR 3.158, 95% CI, 1.047–9.518, *P* = .041), but not the “TP level in CSF > patients’ age” (HR 1.416, 95% CI: 0.513–3.906, *P* = .502) (Table 4).

**Table 4:** Multivariate cox regression analysis associated with the risk of mortality in carcinomatous meningitis.

	HR	95% CI	*P* value
EGFR mutation negative	3.158	1.047–9.518	.041
TP in CSF (>patients’ age)	1.416	0.513–3.906	.502

CSF = cerebrospinal fluid, EGFR = epidermal growth factor receptor, HR = hazard ratio, TP = total protein.

## 4 Discussion

This study demonstrated that the cutoff value of CEA ≥5 ng/ml in CSF is a simple and high-yield diagnostic method for CM diagnosis in patients with lung cancer, but is not a suitable predictive factor for mortality, as demonstrated by Cox regression analysis. Moreover, TP in CSF >patient age might be a novel and/or predictive factor for assessing short-term mortality.

The frequency of brain metastasis in lung cancer patients accounts for up to 20.6% of their clinical course. However, CSF cytology was positive only in 50% to 60% of CM lung cancer patients during the first lumbar puncture,^[3]^ which requires more convenient and useful markers for CM diagnosis. Shi et al^[4]^ described that the 97.5th percentile and maximum value of CSF CEA concentration for 346 patients with non-neoplastic diseases were 0.529 and 2.340 g/dl, respectively.

However, Sudo et al described that CM patients had a median CEA value as high as 42.4 ng/ml in CSF^[5]^ and Twijnstra et al reported that if the cutoff for CEA in CSF was set at 4 ng/ml, the sensitivity and specificity for CM (solid and hematologic tumors) were 31% and 90%, respectively.^[6]^ Similarly, another study demonstrated that the preferred cutoff value of CEA in CSF for CM due to lung cancer, gastric cancer, lymphadenoma, and breast carcinoma was 4.522 g/dl.^[7]^ Although Wang et al reported that the cutoff value of CEA in CSF of 4.7 g/dl has high sensitivity (91.4%) and specificity (91.4%) for discrimination of lung cancer from benign tumor patients,^[8]^ the appropriate cutoff value for CM lung cancer has been scarcely reported.

In this regard, we successfully demonstrated that “CEA in CSF ≥5 ng/ml” has a high diagnostic yield for CM lung patients, but presumably cannot be used as a predictive factor for mortality.

In general, TP levels in CSF depend on the patients’ age: with patients in their 10 s to 40 s having a range from 15 to 45 g/dl, while that of infants and elderly persons seemed to be slightly higher than those in their 10 s to 40 s, both in CM patients and healthy volunteers.^[9]^ There is scarce information regarding age-specific TP in CSF; however, Muller et al^[10]^ reported that the regression line for 296 normal cases can be expressed by the following equations (protein concentration in mg/dl, age in years): “normal CSF protein =23.8 × 0.39 × age ± 15.0.” We considered the equation simply as “TP in CSF >patient age,” a marker for discriminating CM from non-CM patients. This simple marker indicates that if the patient's age is 50, TP in CSF >50 mg/dl reflects high protein levels than healthy adults based on the above equations. In addition, previous reports have described that CM or CM breast cancer patients have high values of TP in CSF greater than 50 mg/dl^[11]^ or 45 mg/dl,^[12]^ while TP levels in the CSF of healthy subjects seemed to be less than their ages.^[10]^ This was consistent with our results that the proportion of “TP levels in CSF >patients’ age” was significantly higher in the CM group than in the non-CM group, which can be a predictive factor for assessing the 90-day survival probability.

With regard to the TCC in CSF, normal data from healthy adults are lacking; however, Conly et al described that normal CSF (non-neoplastic and non-metastatic values) may contain up to 5 WBCs per mm^3^ in adults and 20 WBCs in newborns.^[13]^ Furthermore, Dencker et al reported that the mean TCC values taken from psychiatric patients were higher in males (1.63 cells/μL in the Fuchs-Rosenthal chamber or 1.56 cells/μL in the Jessen chamber) than in females (1.34 cells/μL in Fuchs-Rosenthal chamber or 1.09 cells/μL in Jessen chamber), ^[9]^ which supported our results that patients with TCC ≥7 cells/μL are likely to have CM. With regard to the simple diagnosis for CM, not only “CEA ≧5.0 (ng/ml),” but also a combination of parameters such as “CEA≧5.0 (ng/ml) and TP≧45 (g/dl)” and “CEA≧5.0 (ng/ml) and TCC ≧7 (cells/μL),” could be considered as diagnostic parameters.

In this study, independent factors associated with the risk of mortality were TP in CSF >patient's age, no treatment after diagnosis of CM, and EGFR mutation negative, but not CEA ≥5 ng/ml. Unfortunately, among the predictive factors for mortality, only EGFR mutations seemed to be reliable on multivariate Cox regression analysis, which simply reflects the fact that EGFR mutation-positive patients who received further treatment with TKIs following the initial diagnosis of CM had a longer survival than the negative patients.

The present study has some limitations. It was retrospectively conducted with a relatively small number of patients with CM. However, to the best of our knowledge, there is no report describing whether CEA levels in CSF can be used as a reliable marker for predicting mortality. From this perspective, the present study demonstrated that CEA in CSF ≥5 ng/ml can successfully discriminate between CM lung and non-CM lung patients, but should not be utilized for assessing mortality.

## 5 Conclusion

This study demonstrated that the cutoff values of CEA ≥5 ng/ml, TP ≥45 g/dl, and TCC ≥7 cells/μL are simple and reliable methods for the rapid diagnosis of CM patients with lung cancer, but may not be suitable predictive factors for mortality.

## Author contributions

**Conceptualization:** Yukari Ogawa, Takeshi Saraya.

**Data curation:** Yukari Ogawa, Takeshi Saraya.

**Investigation:** Akinari Noda, Nozomi Kurokawa, Sho Sakuma, Kaori Aso, Sunao Mikura, Miku Oda, Manabu Ishida, Kojiro Honda, Keitaro Nakamoto, Masaki Tamura, Saori Takata, Haruyuki Ishii, Hajime Takizawa.

**Supervision:** Haruyuki Ishii, Hajime Takizawa.

**Writing – original draft:** Yukari Ogawa, Takeshi Saraya.

**Writing – review & editing:** Takeshi Saraya.
